# Evolution of IgE sensitization profiles to *Artemisia* pollen allergens in the allergic pediatric population^☆^

**DOI:** 10.1016/j.waojou.2026.101391

**Published:** 2026-04-29

**Authors:** Jingping Fan, Huifang Song, Chen Zhao, Xiaomin Wang, Qianwei Zhao, Xiaoran Wang, Yumin Wang, Yuan Xia

**Affiliations:** a Inner Mongolia Medical University, Hohhot, 010110, Inner Mongolia, China; b Inner Mongolia Maternity and Child Health Care Hospital, Hohhot, 010020, Inner Mongolia, China

**Keywords:** *Artemisia* pollen allergy, IgE sensitization profiles, Molecular spreading, Pediatric allergic rhinitis

## Abstract

**Objective:**

Allergic rhinitis prevalence has steadily risen among Hohhot's pediatric and adolescent populations. Local epidemiological studies confirm *Artemisia* pollen as the primary aeroallergen causing allergic rhinitis in summer and autumn. This study aimed to investigate how multiple *Artemisia* pollen allergens influence immunological sensitization in children with allergic rhinitis.

**Methods:**

This study systematically investigated IgE sensitization profiles of 196 diagnosed pediatric allergic rhinitis patients with *Artemisia* pollen allergy. Obtained corresponding serum samples, and comprehensive allergen profiling was conducted through enzyme-linked immunosorbent assay and immunoblot analysis. The immunodominant fractions of *Artemisia* pollen allergens were identified using mass spectrometry, with subsequent validation through recombinant allergen purification. Comprehensive pattern analysis revealed distinct sensitization profiles within the study cohort.

**Results:**

This study established *Artemisia* pollen as the predominant summer-autumn aeroallergen in Hohhot, with major allergenic components Art an 7 (69.01%), Art an 4 (56.14%), Art an 1 (18.71%), and Art an 3 (13.45%). Pediatric IgE sensitization profiles demonstrated marked heterogeneity. Monomolecular analysis revealed age-stratified dominance: 94.29% of children aged ≤6 years and 85.71% of those aged 7–14 years exhibited primary sensitization to either Art an 7 or Art an 4. Moreover, both dual- and multi-component sensitization patterns predominantly involved co-recognition of Art an 7 and/or Art an 4.

**Conclusions:**

We propose 2 distinct sensitization trajectories: (A) initial IgE priming by Art an 7 and/or Art an 4 in early childhood, and (B) subsequent molecular spreading to Art an 1 and/or Art an 3 with immunological maturation. These findings established optimal therapeutic window for immune tolerance.

## Introduction

Allergic diseases arise from genetic predisposition and aberrant immune responses to allergens. Respiratory allergy (RA), primarily manifesting as allergic rhinitis (AR) and asthma (AA), exemplifies the “united airway disease” concept due to shared upper/lower airway pathophysiology.^1^ AR is an IgE-mediated reaction triggered by aeroallergens like pollen, dust mites, and animal dander.^2^ Clinically presenting as a symptomatic triad of nasal mucosal edema (congestion), paroxysmal sneezing, and itchy eyes. These symptoms typically persist for more than 60 min per episode and recur on 2 or more consecutive days, thereby fulfilling the temporal diagnostic criteria outlined in the ARIA (Allergic Rhinitis and its Impact on Asthma) guidelines.^3^^,^^4^

AR affects 10–30% of adults and 40% of children globally,^5^ with early onset evidenced by over 5% prevalence in 3-year-olds and over 14% in adolescents.^6^^,^^7^ Its comorbidity with allergic asthma (AA, prevalence 5–10%^8^) reflects shared immunopathology under the “united airway” paradigm. In China, AR/AA prevalence surged dramatically, driven by *Artemisia* pollen sensitization.^4^^,^^9^ Between 2008 and 2018, northern China's *Artemisia vulgaris* sensitization tripled to 30.3%, correlating with expanded cultivation of cross-reactive *Artemisia desertorum* windbreaks.^10^ *Artemisia* pollen dominates autumn aeroallergens in Inner Mongolia,^11^, ^12^, ^13^, ^14^ with regional AR prevalence rates of 4.5% among children aged 6–13 years^15^ and 18.5% in the Inner Mongolia grassland area.^13^

The World Health Organization and International Union of Immunological Societies (WHO/IUIS) Allergen Nomenclature Subcommittee (www.allergen.org) has identified 7 allergens in *Artemisia* pollen.^16^ In China, Art v 1, Art v 3, and Art an 7 are major allergens.^17^ Currently, only Art v 1 and Art v 3 are accessible for component analysis diagnostics (CRDs).^18^ Moreover, emerging evidence suggests that IgE sensitization profiles to *mugwort* pollen allergens exhibit familial aggregation, with significant concordance observed between Chinese parents and their offspring, further underscoring the importance of understanding component-resolved sensitization patterns in pediatric populations.^19^ Despite rising pediatric AR cases, *Artemisia* sensitization research in Inner Mongolia remains adult-focused. This study investigates multiple allergenic proteins' immunological effects on children's sensitization profiles and their molecular spreading patterns, aiming to improve diagnostic accuracy and advance personalized immunotherapy.

## Methods

### Allergic pediatric subjects

Patients with allergic symptoms were tested at Inner Mongolia Maternity and Child Health Care Hospital during August–September 2021–2022 without specialized recruitment. All these patients have been confirmed to have AR with or without AA based on the guideline for diagnosis and treatment of pediatric allergic rhinitis and guideline for the diagnosis and optimal management of asthma in children,^20^ with non-atopic children as controls. All patients have the following characteristics: (a) presence of 2 or more local symptoms (nasal obstruction, rhinorrhea, nasal pruritus, or paroxysmal sneezing) with daily symptom duration exceeding 1 h; (b) *Artemisia* pollen allergen-specific IgE (sIgE) antibodies (sIgE ≥0.35 IU/mL); (c) with or without AA, defined as a history of dyspnea, wheezing, and/or coughing episodes, and confirmed by a positive bronchial dilation test; (d) aged 0–14 years.

The study was approved by the ethics committee of the Inner Mongolia Maternity and Child Health Care Hospital (No.2020064) and informed consent was obtained from all patients' parents or guardians prior to serum collection.

### Total and specific IgE

A retrospective analysis of serum sIgE levels was conducted using the Allergen-Specific IgE Antibody Detection Kit (HOB Biotech, Suzhou, China) in Hohhot, Inner Mongolia, China. This kit is used to detect total IgE antibodies and allergen-specific IgE antibodies in serum by enzyme-linked immunosorbent assay (ELISA) rapid test strip technology, with each strip containing: 1 positive control, 1 negative control, 1 total IgE detection zone, and 13 allergen-specific IgE panels. The allergens included House dust mite (House dust mite, *Dermatophagoides pteronyssinus*), Tree pollen (Cypress, Elm, Sycamore, Willow, Poplar), *Artemisia* pollen (Ragweed, *Artemisia*, Wormwood), Mold (*Penicillium notatum*, *Aspergillus fumigatus*, *Cladosporium*, *Alternaria*, *Rhizopus*, *Mucor*), Animal dander (Dog, Cat), Milk, Egg fractions, Marine fish (Cod, Salmon, Bass), Shellfish (Shrimp, Crab, Scallop), Fruit (Mango, Pineapple, Apple, Peach, Strawberry), Nut (Peanut, Pistachio, Cashew, Hazelnut), Beef and Lamb.

According to the Clinical Study Guidelines for Allergen-Specific IgE Antibody Test Kits, rate the reactivity of the sIgE tests as: class 0 (<0.35 IU/mL), class 1 (≥0.35 and < 0.70 IU/mL), class 2 (≥0.70 and < 3.50 IU/mL), class 3 (≥3.50 and < 17.50 IU/mL), class 4 (≥17.50 and < 50.00 IU/mL), class 5 (≥50.00 and < 100.00 IU/mL), and class 6 (≥100.00 IU/mL). The serum sIgE level for pollen above class 0 was considered positive.

### Extraction of pollen protein

The pollen of *Artemisia annua* was collected in the suburbs of Hohhot city, Inner Mongolia, during the flowering season. The pollen samples were fully ground with liquid nitrogen, dehydrated with acetone, and then stored at −20 °C for reserve. The pollen proteins were extracted using the Pollen Protein Extraction Kit (Bestbio, Shanghai, China).

### Purification and recombinant expression of *Artemisia* pollen components

To construct expression plasmids for the 4 Artemisia pollen allergens, their respective mRNA sequences were first comprehensively identified and optimized. These sequences were cloned into the pFastBac1 vector to generate recombinant plasmids containing both a His-tag and a Flag-tag. The plasmids were transformed into DH10Bac competent cells (General Biologicals, Anhui, China) via ice incubation for 30 min, heat shock at 42 °C for 90 s, and subsequent addition of 500 μL SOC medium (composition: 2% (w/v) tryptone, 0.5% (w/v) yeast extract, 0.05% (w/v) NaCl, 2.5 mM KCl, 10 mM MgCl_2_, 20 mM glucose). After 4 h incubation, cells were plated on triple-antibiotic LB agar containing 7 μg/mL gentamicin, 100 μg/mL kanamycin, 10 μg/mL tetracycline, 40 μg/mL IPTG, and 40 μL 2% X-gal. Plates were inverted and incubated at 37 °C for 48 h.

White colonies identified via blue-white screening were selected and inoculated into 3 mL of SOC medium supplemented with the appropriate antibiotic. The cultures were incubated overnight at 37 °C with shaking at 200 rpm to facilitate bacterial growth and plasmid amplification. Bacmids were extracted using the M5 HiPer Plasmid Miniprep Kit (Mei5Bio, Beijing, China) and stored at −20 °C. Recombinant bacmids were transfected into Sf9 insect cells using FuGENE® HD (Promega, Shanghai, China) to generate P1 baculovirus, which was amplified to produce high-titer P2 stocks.

Sf9 cells were harvested by centrifugation (4000 rpm, 10 min, 4 °C), resuspended in lysis buffer (20 mM Tris-HCl, 150 mM NaCl, 1 mM PMSF, 10 mM imidazole, pH 8.0), and lysed by sonication (300 W, 3 s on/5 s off, 30 min total). Lysates were clarified by centrifugation (18,000 rpm, 15 min, 4 °C), followed by purification of Art an 1, Art an 3, Art an 4, and Art an 7 allergens using Ni-IDA beads (Smart-Lifesciences, Changzhou, China) per manufacturer instructions. SDS-PAGE analysis was performed on samples collected from each purification step to evaluate the purity of the target protein.

### Western blotting

The protein concentration for all extracts and purified allergen components was estimated using the BCA Protein Assay kit (Beyotime, Shanghai, China) with bovine serum albumin as a standard. All protein samples were diluted to the same total protein concentration.

The samples were loaded on 12.5% SDS-PAGE gels for protein separation and subsequently electroblotted onto PVDF membranes (Beyotime, Shanghai, China). After blocking of excess protein-binding sites with QuickBlock™ Blocking Buffer (Beyotime, Shanghai, China) for 30 min, the PVDF membranes were incubated with the primary antibody (patient serum samples, 1:40–1:300 dilution) at 4 °C overnight. After TBST washing, the secondary goat anti-human IgG-HRP antibody (ABclonal, Wuhan, China) was added for 1 h incubation. Lastly, immunoreactions were visualized by the ECL kit (Affinity Biosciences, Changzhou, China). Densitometric analyses were performed using the GelView 6000Plus system (Tanon Technology Co., Ltd., Shanghai, China).

### Statistical analysis

Recombinant plasmids for *Artemisia* pollen allergens were constructed using SnapGene 4.2.4, with data plotting performed using Origin 2021 and R 4.3.3. Statistical analyses were performed using SPSS 25.0 and R 4.3.3, with a value of *P* < 0.05 considered as significantly different. Continuous variables following normal distribution were expressed as mean ± standard deviation ($\bar{x}$ ± s), while non-normally distributed data were reported as median and interquartile range. For two-group comparisons: independent t-tests were applied to normally distributed variables, and Wilcoxon rank-sum tests to non-parametric data. Categorical variables were compared using the chi-square test or Fisher's exact test. Multi-group comparisons with normal distribution were analyzed by ANOVA, followed by pairwise post-hoc testing with the LSD method.

## Results

### Demographic and clinical characteristics of the study participants

A total of 196 children (123 males and 73 females) were enrolled in this study (Table 1). Participants aged 2–14 years (mean 5.1 ± 2.6 years) were stratified into 3 age groups: 63 (32.14%) aged 0–3 years, 84 (42.86%) aged 4–6 years, and 49 (25.00%) aged 7–14 years. Comorbid allergic conditions included 31 cases (15.82%) of allergic conjunctivitis, 9 (4.6%) of urticaria, and 7 (3.6%) of eczema. Based on clinical diagnosis, 153 (78.06%) participants were categorized into AR and 43 (21.94%) into the AA. No statistically significant difference was observed in the prevalence of children across different genders and age groups (*P* > 0.05). Complete demographic characteristics are presented in Supplementary Table S1.

**Table 1:** Demographics and characterization of the study population in Hohhot.

Characters	AR (n = 153)	AA (n = 43)	*P-*value
Gender, no. (%)			
Males	92 (60.13)	31 (72.09)	0.152
Females	61 (39.87)	12 (27.91)	
Age, no. (%)			
≤3	54 (35.30)	9 (20.93)	0.115
4–6	65 (42.48)	19 (44.19)	
7–14	34 (22.22)	15 (34.88)	
Total IgE, median, IQR (IU/mL)	142.38 (101.37–265.93)	195.57 (109.93–500.00)	0.070
*Artemisia*-sIgE, median, IQR (IU/mL)	39.67 (7.86–102.53)	71.57 (40.99–200.00)	0.024^a^
Allergen positive no. (%)			
Tree pollen (Cypress, Elm, Sycamore, Willow, Poplar)	78 (50.98)	25 (58.14)	0.406
Mold (*Penicillium notatum, Aspergillus fumigatus, Cladosporium, Alternaria, Rhizopus, Mucor*)	40 (26.14)	15 (34.88)	0.260
Animal dander (Dog, Cat)	38 (24.84)	9 (5.88)	0.596
House dust mite (House dust mite, *Dermatophagoides pteronyssinus*)	1 (0.65)	1 (2.33)	0.392
House dust	10 (6.54)	3 (6.98)	1.000
Milk	28 (18.30)	4 (9.30)	0.158
Beef and lamb	7 (4.58)	0 (0.00)	0.351
Egg fractions	8 (5.23)	1 (2.33)	0.687
Nut (Peanut, Pistachio, Cashew, Hazelnut)	2 (1.31)	3 (6.98)	0.071
Fruit (Mango, Pineapple, Apple, Peach, Strawberry)	1 (0.65)	2 (4.65)	0.122
Shellfish (Shrimp, Crab, Scallop)	2 (1.31)	0 (0.00)	1.000
Marine fish (Cod, Salmon, Bass)	2 (1.31)	1 (2.33)	0.526
sIgE of *Artemisia* pollen, no. (%)			
Class 1	14 (9.15)	5 (11.63)	0.007^b^
Class 2	19 (12.42)	2 (4.65)	
Class 3	24 (15.69)	1 (2.32)	
Class 4	26 (16.99)	3 (6.98)	
Class 5	32 (20.91)	16 (37.21)	
Class 6	38 (24.83)	16 (37.21)	
Sensitized to *Artemisia* component, n (%)			
Art an 1	22 (14.37)	10 (23.26)	0.164
Art an 3	20 (13.07)	3 (6.98)	0.273
Art an 4	79 (51.63)	17 (39.53)	0.161
Art an 7	89 (58.17)	29 (67.44)	0.272

Abbreviations: IgE, Immunoglobulin E; IQR, InterQuartile Range; sIgE, specific IgE antibody.

a *P* < 0.05

b *P* < 0.01

### Specific IgE profiles to allergens

The median serum total IgE level among participants was 166.47 IU/mL (InterQuartile Range [IQR], 101.96–304.17), with *Artemisia* pollen-specific IgE (sIgE) levels at 51.79 IU/mL (IQR,10.39–117.01). Children diagnosed with AA demonstrated significantly higher serum levels of *Artemisia* pollen-sIgE compared to those with AR alone (*P* = 0.024) (Table 1).

**Fig. 1:**
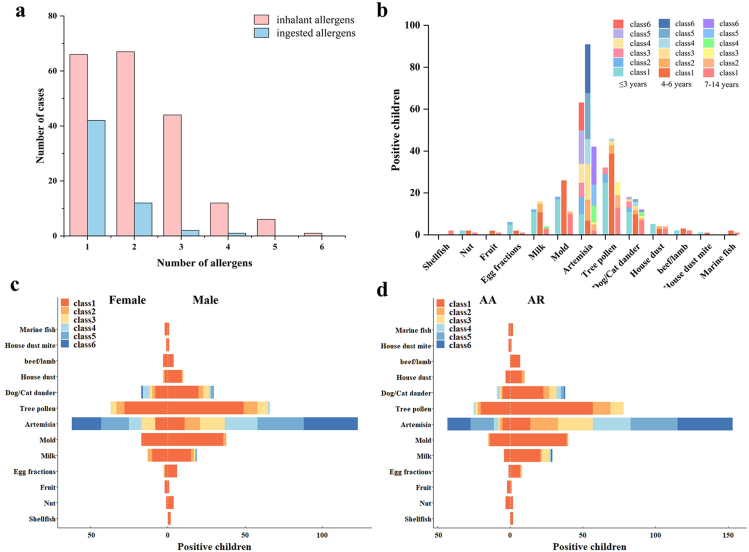
Distribution of allergens sensitization among children. (a) Sensitization of children's sensitization to different number of allergens. (b) Prevalence of allergen sIgE-positivity among age groups: <3, 4–6, and 7–14 years old. (c) Prevalence of allergen sIgE-positivity among gender groups. (d) Prevalence of allergen sIgE-positivity among AR and AA groups

While all 196 children exhibited sensitization to *Artemisia* pollen allergens, 66.33% (130/196) showed co-sensitization to other inhalant allergens, primarily tree pollen (52.04%, 102/196), mold (28.06%, 55/196) and dander (dog and cat) (23.98%, 47/196). 42 cases (21.43%, 42/196) were positive for ingested allergens, with milk (16.33%, 32/196), egg fractions (4.59%, 9/196) and beef and lamb (3.57%, 7/196) being the top 3. The 102 (66.67%, 102/153) children with AR were positive for other inhalant allergens, and 33 (21.57%, 33/153) were positive for ingested allergens. Relatively, the 28 (65.12%, 28/43) children with AA were positive for other inhalant allergens and 9 (20.93%, 9/43) were positive for ingested allergens (Fig. 1a). There was no statistically significant difference observed in the rate of positivity for each allergen among children of different age and gender groups, or in the AA and AR groups (*P* > 0.05) (Supplementary Table S2).

The sensitization profile analysis demonstrated a graded distribution of *Artemisia*-specific IgE responses, with proportions progressively increasing from class 1 (9.69%, n = 19) to class 6 (27.55%, n = 54). Notably, children with higher sensitization grades (classes 5–6) collectively constituted 52.04% (102/196) of the study cohort, exceeding the cumulative proportion of lower grades (classes 1–4, 47.96%). A statistically significant difference was observed between different sIgE classes (*χ*^*2*^ = 14.855, *P* = 0.007). Notably, the 4-6-year age group demonstrated the highest *Artemisia* pollen sensitization rate. In children aged 7–14 years, high-grade sensitization (sIgE classes 4–6) predominated over lower grades (classes 1–3) (Table 1, Fig. 1b). Both genders showed comparable patterns, with *Artemisia* pollen-sensitized clustering in classes 5–6 (Fig. 1c). In the AA and AR groups, the prevalence of high *Artemisia* sIgE level was higher in the AR group, and the sensitization increased with the severity of the sIgE level. The proportion of children with *Artemisia* sIgE level of class 5–6 was significantly higher than classes 1–4 in the AA group (Fig. 1d).

### Patterns of sensitization to *Artemisia* allergen components among those sensitized to *Artemisia* pollen

**Fig. 2:**
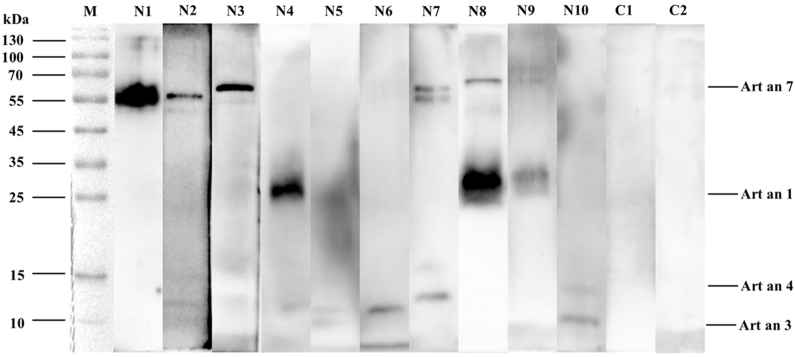
Immunoblots of individual serum with *Artemisia* pollen protein. *Artemisia* pollen proteins were incubated separately with 10 serum samples, 10 positive and 2 negative

To characterize the allergenic profile of *Artemisia* pollen within the studied population, immunoblot analysis revealed 4 distinct IgE-reactive protein bands at molecular weights of approximately 65, 28, 15, and 10 kDa (Fig. 2). Subsequent identification via mass spectrometry, aligned with the WHO/IUIS Allergen Nomenclature database, confirmed these proteins as Art an 7, Art an 1, Art an 4, and Art an 3, respectively. Component-resolved diagnostics demonstrated a hierarchical pattern of IgE reactivity: Art an 7 exhibited the highest sensitization rate at 59.7% (118/196), followed by Art an 4 at 49.0% (96/196), Art an 1 at 16.3% (32/196), and Art an 3 at 11.7% (23/196). These findings underscore the predominance of Art an 7 and Art an 4 as major allergens in this cohort, highlighting their relevance in the molecular diagnosis and management of *Artemisia*-induced allergic responses.

**Fig. 3:**
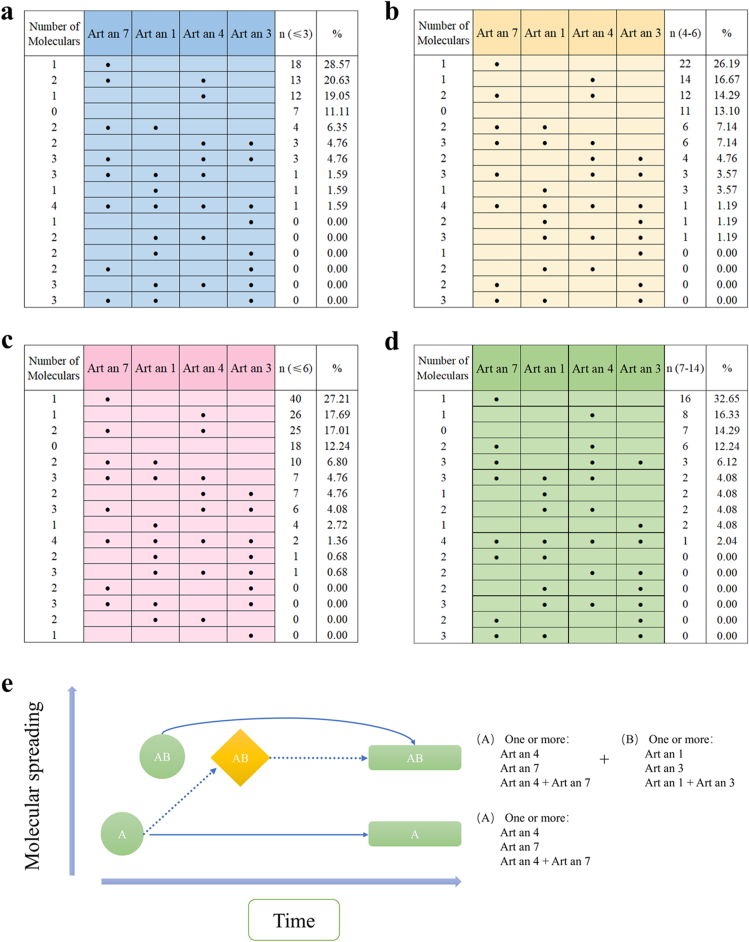
IgE sensitization profiles to 4 *Artemisia* pollen components in 196 *Artemisia*-sIgE-positive subjects. (a) 0–3 years. (b) 4–6 years. (c) ≤6 years. (d) 7–14 years. (e) Pattern of sensitization processes in *Artemisia* pollen allergenic components. Each line denotes 1 profile (ordered by decreasing frequency), with absolute frequencies shown on the right

However, 11.2% (25/196) of subjects showed no detectable IgE reactivity to these components (Table 1). Notably, 50.0% (98/196) of participants demonstrated a single positive component, 26.0% (51/196) demonstrated 2 positive components, 9.7% (19/196) displayed 3 positive components, and only 1.5% (3/196) of children exhibited 4 positive components. In almost all children, the initial IgE response is directed against Art 7, a sort of initiatory molecule of this IgE response. Sensitization started against Art an 7 and/or Art an 4 (defined as group A molecules), then involved Art an 1 and Art an 3 (group B molecules). This progression could be described as the AB march of *Artemisia* allergy (Fig. 3).

When the data were stratified by age, it was found that 47.9% of children aged 6 years and younger responded to only 1 molecule (monomolecular profile), 29.3% responded to 2 molecules (oligomolecular profile), and 10.9% responded to 3 or more molecules (polymolecular profile) (Fig. 3c). A comparable pattern emerged among children aged 7–14 years, with 57.1% responding to only 1 molecule, 16.3% responding to 2 molecules, and 12.2% responding to 3–4 molecules (Fig. 3d). All of these children demonstrated a predominant sensitivity to Art an 7 or Art an 4. The oligomolecular pattern in children under 3 years of age was predominantly characterized by “Art an 7-Art an 4” (20.63%, 13/63), with a minor proportion exhibiting “Art an 7-Art an 1” (6.35%, 4/63) or “Art an 4-Art an 3” (4.76%, 3/63) sensitization patterns (Fig. 3a). In contrast to children aged 4–6 years, the proportion of monomolecular sensitization decreased, while the proportion of sensitization to 3 allergenic molecules increased, predominantly manifesting as a “Art an 7-Art an 4-Art an 1” pattern (Fig. 3b). By the age of 7, the proportion of sensitization to the 3 allergenic components decreased. Post this age, the oligomolecular sensitization pattern predominantly manifested as “Art an 7-Art an 4” (12.24%, 6/49), with smaller proportions exhibiting “Art an 4-Art an 1” (4.08%, 2/49) and “Art an 7-Art an 4-Art an 1” (4.08%, 2/49). Notably, the prevalence of the “Art an 7-Art an 4-Art an 3” sensitization pattern declined with age (Fig. 3d).

**Fig. 4:**
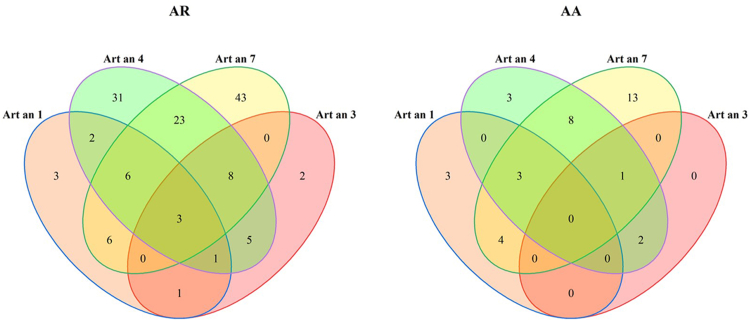
Venn diagrams of the interrelation of sensitized allergens in AR and AA groups

A further finding of interest is that Art an 4 and Art an 3 tend to occur together, while Art an 7 and Art an 3 rarely co-occur (Fig. 3e). Moreover, for both groups, the relationship between sensitizing allergens and symptoms was examined. Patients with AR had a higher percentage of reactions to Art an 3, Art an 4, and Art an 7 than patients with AA (Fig. 4).

### Purified allergen analysis

**Fig. 5:**
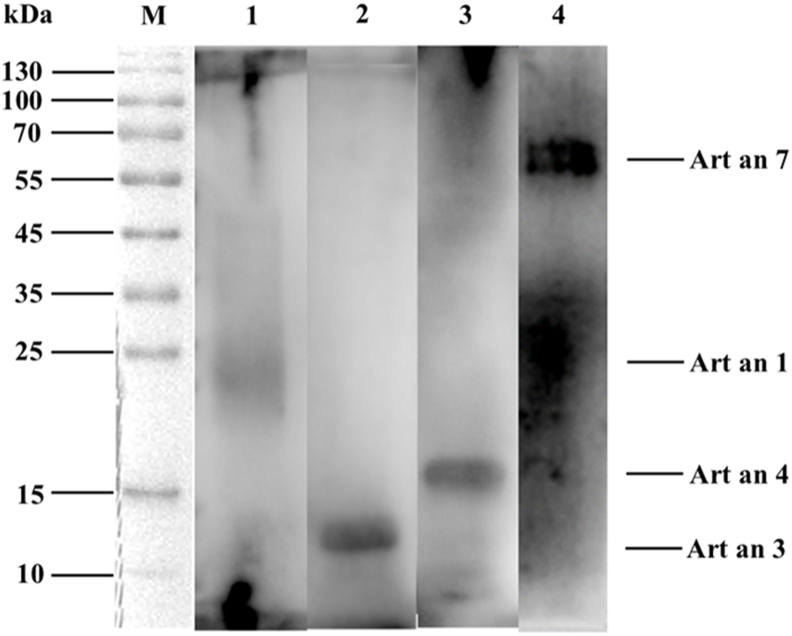
Immunoblot of recombinant proteins using serum from *Artemisia*-allergic subjects. Lane M, standard marker; Lane 1–4 represented purified recombinant allergens of Art an 1, Art an 3, Art an 4, and Art an 7 respectively

The recombinant *Artemisia* pollen allergenic proteins were obtained by following the method described in the material. The amino acid sequences of the purified recombinant proteins were validated by mass spectrometry. Cross-referencing with the WHO/IUIS Allergen Nomenclature database confirmed that these proteins correspond to Art an 7, Art an 1, Art an 4, and Art an 3, respectively. Following separation by SDS-PAGE (Supplementary Figs. S1–S4), IgE binding to purified recombinant allergens were investigated by immunoblotting using serum from *Artemisia*-allergic patients (Fig. 5). All recombinant *Artemisia* pollen allergenic proteins were observed to exhibit distinct bands at their corresponding positions, consistent with the predicted protein size and exhibiting expression levels comparable to that of *Artemisia* pollen protein extracts. These findings suggest that they possess similar immunogenicity.

## Discussion

In our cohort of 196 pediatric AR patients, 21.94% had comorbid AA, and 15.82% had AC, consistent with epidemiological patterns where 70–80% of AR cases manifest as isolated disease and 20–30% are associated with comorbid asthma.^21^ The AA-AR association suggests shared allergic airway mechanisms.^11^ Bousquet et al^22^ proposed that the ‌multimorbid allergic phenotype‌ (AA, AR, and AC) may synergistically increase disease burden. Our data didn't reveal ‌significant gender-based disparities‌, with male subjects ‌predominating‌ in both the AR (60.13% vs 39.87%) and AA (72.09% vs 27.91%) cohorts (*P* = 0.152).

Furthermore, ‌age-stratified analysis‌ demonstrated that the 4–6 years subgroup comprised of ‌largest demographic proportion‌ (42.86%) compared to other developmental stages (*χ*^*2*^ = 4.33, *P* = 0.115), suggesting potential age-dependent immunological susceptibility patterns. Consistent with these findings, a nationwide epidemiological survey by Guo et al^23^ revealed ‌significantly elevated asthma prevalence‌ in preschool children (4–6 years) compared to other pediatric age strata. Additionally, following the findings of Luo et al,^24^ the prevalence of *Artemisia* sensitization increased with age in the higher age groups. In this study, the *Artemisia* pollen sensitization rate demonstrated an age-dependent epidemiological pattern, peaking in children aged 4–6 years. Notably, within the school-aged cohort (7–14 years), high-grade *Artemisia* sIgE reactivity (classes 4–6) exhibited significant quantitative predominance over low-grade sensitization (classes 1–3) (72% vs 28%), with the proportion of severe sensitization cases (≥class 4) showing elevated prevalence compared to both 0–3 years and 4–6 years age groups. This age-dependent susceptibility pattern was further verified by Wu et al,^25^ whose ‌quantitative aeroallergen sensitization analysis‌ demonstrated that 7–14 years exhibited ‌enhanced sIgE responsiveness‌ (*P* < 0.05 for each) to common airborne allergens (cat and dog) relative to younger children (0–6 years). This age-dependent susceptibility pattern reflects enhanced sIgE responsiveness to aeroallergens in older children, potentially due to increased outdoor exposure.

IgE quantification's diagnostic role in atopic disorders is confirmed by current guidelines (EAACI/AAAAI 2022).^26^ Both total and *Artemisia* sIgE levels demonstrated statistically significant elevation (*P* = 0.024) in children with AA compared with AR. In both pediatric cohorts with AR and AA, we observed significant inhalant allergen sensitization rates, with 71.43% of patients exhibiting multi-allergen sensitization (≥2 allergens). 52.04% of the children demonstrated co-sensitization to *Artemisia* pollen and tree pollen. The secondary allergens are molds (28.06%) and the dander of dogs and cats (23.98%), with minimal to no allergy to house dust mites. These findings contrasted with those of Ma et al,^11^ who reported that children more frequently showed a monosensitization pattern. However, it was consistent with the poly-sensitization patterns of AR patients who were sensitized to 2 or more pollens (79.4%) (including *Artemisia*, *Chenopodium*, and *Humulus scandens*) in northern China.^13^ Conversely, only 21.43% of the children exhibited an allergy to ingestible allergens, predominantly to milk (16.33%), egg components (4.59%), and beef and lamb (3.57%). As reported by Li et al,^27^ the prevalence of food allergies in Beijing was found to exceed 20%, with a particular prevalence of allergies to grass pollen and tree pollen.

The IgE-binding activity of *Artemisia* pollen components was assessed via IgE immunoblotting using serum samples from pediatric participants. Subsequently, the 4 recombinant *Artemisia* allergen proteins were cloned, expressed, and characterized. Immunodominant IgE-reactive components were identified as follows: Art an 1 (16.3%), Art an 3 (11.2%), Art an 4 (49.0%), and Art an 7 (59.7%). Among 100 *mugwort*-sIgE positive patients in western China, the positivity rates for Art v 1, Art v 3, and Art v 4 were 52.0%, 41.0%, and 31.0%, respectively.^28^ In northwestern China, among 135 patients with *Artemisia* allergy, the positivity rates for the *Artemisia desertorum* allergens Art d 1, Art d 3, and Art d 7 were 85.2%, 88.9%, and 82.2%, respectively. The number of allergens and the level of specific IgE to Art d 3 were independently associated with asthma.^29^ Gao et al^17^ reported significant differences in sensitization rates to Art v 1 (84%), Art an 7 (87%), Art v 3 (66%), and Art ar 2 (48%) among children, teenagers, and adults. Specifically, the adult group demonstrated significantly lower serum IgE levels to *mugwort* and Art an 7 than the child and teenage groups. The discrepancy in these findings indicates that children with AR in Hohhot may exhibit a distinct component sensitization profile compared to adult AR patients, with age likely serving as the principal factor underlying this difference.

Art an 7, formally characterized as a galactose oxidase by the IUIS Allergen Nomenclature Subcommittee, demonstrated IgE sensitization rates of 73–92% in *Artemisia* pollen-allergic cohorts across European epidemiological studies during 1990–2008^30^, ^31^, ^32^, ^33^, and more than 94% of *Artemisia* pollen-sensitized patients exhibit specific IgE reactivity to recombinant Art an 7.^32^ This allergen has been mechanistically linked to *mugwort*-food cross-reactivity through structural homology with galactose-containing glycoproteins.^34^ Art an 4, officially designated a profilin family allergen, demonstrates greater than 70% sequence identity and preserved actin-binding domains with pan-allergenic profilins,^35^ presenting a sensitization prevalence of 34–36% in *mugwort* allergic patients.^30^^,^^36^ This structural conservation underlies its cross-reactive IgE epitopes, as evidenced by immunoblot inhibition when pre-incubated with Bet v 2 or Man i 4 IgE-positive sera.^37^ Art an 1 is a two-domain glycoprotein comprising an N-terminal defensin-like domain stabilized by 3 conserved disulfide bonds and a C-terminal hydroxyproline-rich glycodomain. It is established as a diagnostic marker allergen for genuine *mugwort* pollen sensitization.^38^ Epidemiological studies demonstrate that up to 41% of European/North American patients with pollen allergy exhibit sensitization to Art an 1 confirmed by component-resolved diagnosis.^26^^,^^39^ Art an 3, an immunodominant non-specific lipid-transfer protein (nsLTP), is recognized as the prototype allergen of the nsLTP family, which is one of the important allergies in adults in the Mediterranean region. About 9% of more than 23,000 allergy patients in the central and southern regions of Italy are sensitive to nsLTP, much higher than in the northern regions.^40^^,^^41^ About 12% of 2000 Spanish pollen allergy patients were found to be sensitive to nsLTP,^42^ and there were differences in sensitivity to nsLTPs among seasonal allergy patients in different regions.^43^ Geographical and climatic factors may also cause differences in the prevalence of nsLTP allergies in different countries.^44^

Children with AR in this region showed different sensitivities to these 4 allergen components. This phenomenon may be related to the natural abundance of different components in pollen. Morales et al^45^ demonstrated that Profilins are greatly expressed near the pollen tube, making them highly abundant in pollen, resulting in the actual exposure dose varying between different children. On the other hand, current molecular spreading theory postulates that primary sensitization to an initiating allergen molecule during a critical developmental window induces progressive IgE epitope spreading, ultimately leading to extreme heterogeneity in the IgE sensitization spectrum in allergic populations. This process manifests as distinct sensitization trajectories: monomolecular (1 allergen), oligomolecular (2–4 allergens), or polymolecular (≥5 allergens) profiles, governed by individual atopic endotypes and cumulative allergen exposure dose.^46^

In this study, sensitization patterns varied across age groups, with the monomolecular profile (sensitization to a single allergen) being the most prevalent, followed by the oligomolecular profile (sensitization to 2 allergens). In contrast, the polymolecular profile (sensitization to 3 or more allergens) exhibited significantly lower occurrence rates. The oligomolecular pattern in children under 3 years of age was predominantly “Art an 7-Art an 4” (20.63%), the proportion of monomolecular profile decreased in children between 4 and 6 years of age (46.4%), and the proportion of sensitization to 3 allergens increased, with a predominantly “Art an 7-Art an 4-Art an 1” pattern (7.14%), while children aged 7–14 years showed a decrease in sensitization to 3 allergenic molecules, with the predominant polymolecular profile again being “Art an 7-Art an 4” (12.24%). Art an 7 or Art an 4 sIgE was detected in the sera of most of the children included in the present study, and both single and multimolecule sensitized children showed co-sensitization to Art an 7 and/or Art an 4.

Thus, Art an 7 and/or Art an 4 may be the initiating molecules for the IgE response to pratense pollen in children in this region (defined as group A), which further extends to Art an 1 and/or Art an 3 in a subset of children with increasing age (defined as group B), showing “Art an 7-Art an 4-Art an 1”, “Art an 7-Art an 4-Art an 3”. The AB process pattern of sensitization to *Artemisia* allergens in children was delineated, representing a molecular spreading trajectory that has been reported in several previous studies. A national cross-sectional survey conducted by the Italian Pediatric Allergy Network (IPAN) demonstrated that specific IgE to Phl p 1, Phl p 7, and Phl p 12 can serve as reliable biomarkers for the diagnosis of *Phleum pratense* pollen allergy in pediatric populations. Moreover, the IgE sensitization profiles among affected children exhibited marked heterogeneity The IgE response to *Phleum pratense* pollen in children is initiated by Phl p 1 (the initiating molecule), with the production of Phl p 1 IgE in serum generally preceding the onset of clinical symptoms by several years. This is followed by a gradual expansion to molecules such as Phl p 2 and Phl p 4 at the onset of the disease, and ultimately covering Phl p 5, Phl p 6, and Phl p 11 after the exacerbation of symptoms. Furthermore, it is postulated that the initial phase of molecular diffusion (eg, the monomolecular or oligomolecular sensitization stage) represents the optimal juncture for immune modulation.^47^ A notable similarity exists between the molecular processes involved in the context of *Dermatophagoides pteronyssinus* sensitization and those involved in the context of allergies to *Phleum pratense* pollen, as evidenced by molecular spreading. In particular, the Der p 1, Der p 2 and Der p 23 allergens have been identified as playing a pivotal role in the initiation of this process, specifically in the initial stage (defined as group A), which is followed by the progression to the Der p 4, Der p 5, Der p 7 and Der p 21 (defined as group B) allergens, and ultimately, to the Der p 11, Der p 14, Der p 15 and Der p 18 (defined as group C) allergens. This progression pattern has been designated the ABC progression model of house dust mite allergy. Participants exhibiting the most extensive ABC IgE sensitization stage demonstrated a significantly increased risk of developing mite-associated AR and AA compared to those with monomolecular sensitization.

Furthermore, IgE sensitization to initiator molecules in otherwise healthy children at ≤5 years of age was predictive of asthma development during school age. IgE sensitization in healthy children to initiatory molecules at age 5 years or younger predicted the development of asthma at school age.^46^ Consequently, IgE tests employing individual allergenic molecules are regarded as a more precise and informative option than those based on allergenic extracts, particularly for patients with polysensitization.

In conclusion, this study characterized *Artemisia* sensitization patterns in AR children from Hohhot, identifying 4 major allergens: Art an 7, Art an 1, Art an 4, and Art an 3. We observed a molecular spreading trajectory where Art an 7 and/or Art an 4 typically initiate IgE responses, with subsequent progression to Art an 1 and/or Art an 3 in older children. These findings reveal cross-reactive *Artemisia* components and demonstrate that early detection of this sensitization pattern enables timely immunoprevention and immunotherapy during the critical immune tolerance window.

## Abbreviations

RA, respiratory allergy; AR, allergic rhinitis; AA, allergic asthma; ARIA, Allergic Rhinitis and its Impact on Asthma; WHO, World Health Organization; IUIS, International Union of Immunological Societies; CRDs, component analysis diagnostics; IgE, Immunoglobulin E; sIgE, specific IgE antibody; PVDF, Polyvinylidene fluoride; TBST, Tris-buffered saline with tween; ECL, enhanced chemiluminescence; AC, allergic conjunctivitis; IQR, InterQuartile Range; EAACI, European Academy of Allergy and Clinical Immunology; AAAAI, American Academy of Allergy, Asthma and Immunology; Art, Artemisia; Bet, Betula verrucosa; Man, Mangifera indica; nsLTP, non-specific lipid-transfer protein; IPAN, Italian Pediatric Allergy Network; Phl, Phleum pratense; Der, Dermatophagoides pteronyssinus.

## Data availability statement

The data supporting the findings of this study are available from the corresponding author upon reasonable request.

## Author contributions

Jingping Fan, Chen Zhao, and Yuan Xia conceived and designed the study. Huifang Song and Yumin Wang were involved in the clinical study of patients and conducted IgE test. Chen Zhao, Xiaomin Wang, and Yuan Xia collected pollens and performed the protein purification. Jingping Fan, Chen Zhao, Xiaomin Wang, Qianwei Zhao, and Xiaoran Wang performed Western Blot experiments and data analysis. Jingping Fan, Huifang Song, and Chen Zhao drafted the manuscript in close collaboration with all co-authors. Yumin Wang and Yuan Xia were joint corresponding authors.

## Ethics approval

The study was approved by the ethics committee of the Inner Mongolia Maternity and Child Health Care Hospital (No.2020064) and informed consent was obtained from all patients' parents or guardians prior to serum collection.

## Authors’ consent for publication

All authors have reviewed and approved the final version of the manuscript and agree to its submission for publication.

## Declaration of generative artificial intelligence (AI) and AI-assisted technologies in the writing process

Nothing to disclose.

## Funding

This study was partly supported by the Science and Technology Planning Project of Inner Mongolia Autonomous Region of China (Grant nos. 2021GG0191), Natural Science Foundation of Inner Mongolia Autonomous Region of China (2024MS08034), Natural Science Foundation of Inner Mongolia Medical University of China (YKD2023MS029), Program for Young Talents of Science and Technology in Universities of Inner Mongolia Autonomous Region (NJYT22012), Science and Technology Program of the Joint Fund of Scientific Research for the Public Hospitals of Inner Mongolia Academy of Medical Sciences (2024GLLH0198).

## Declaration of competing interest

The authors have no conflicts of interest to report.
