# Supplementary material for: Evolution of IgE sensitization profiles to Artemisia pollen allergens in the allergic pediatric population
Source: World Allergy Organ J. 2026 Apr 29;19(5):101391. doi: 10.1016/j.waojou.2026.101391 (PMC13141757; doi:10.1016/j.waojou.2026.101391)
Supplement: Multimedia component 1 [file mmc1.pdf]

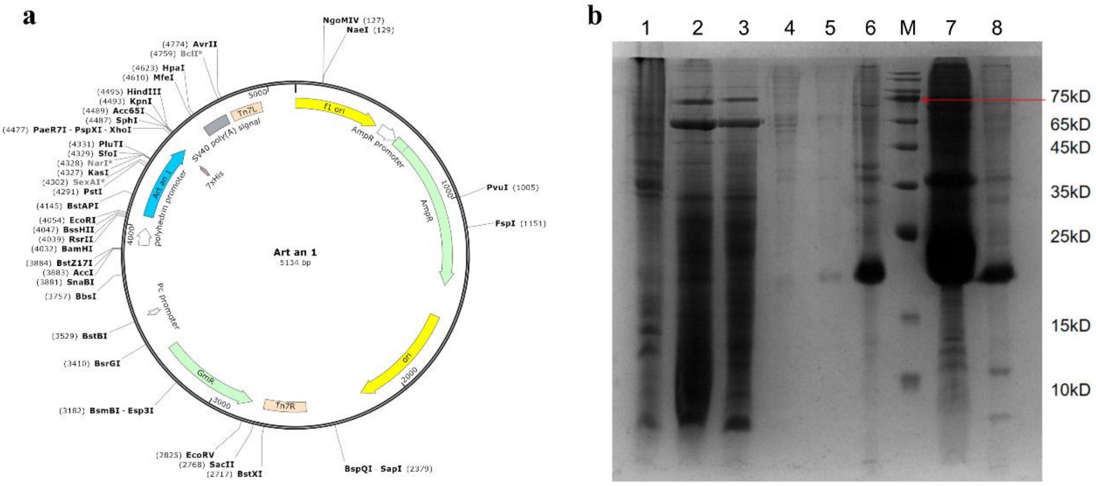

Fig. S1 Purification of Art an 1 from *Artemisia* pollen extract. (a) pFastBac-Art an 1 recombinant plasmid. (b) Affinity Chromatography analysis of recombinant protein Art an 1. Lane M, standard marker; Lane 1: cell lysate precipitate; Lane 2: cell lysate supernatant; Lanes 3-5: washes with 20 mM, 40 mM, and 60 mM imidazole, respectively; Lane 6: nickel-affinity chromatography filler; Lanes 7-8: eluates with 300 mM and 500 mM imidazole, respectively.

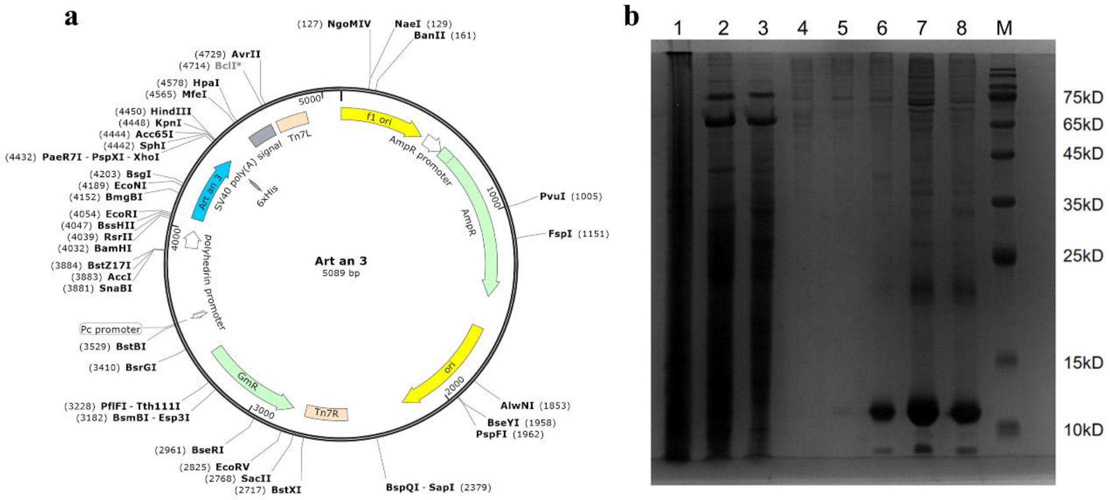

Fig. S2 Purification of Art an 3 from *Artemisia* pollen extract. (a) pFastBac-Art an 3 recombinant plasmid. (b) Affinity Chromatography analysis of recombinant protein Art an 3. Lane M, standard marker; Lane 1: cell lysate precipitate; Lane 2: cell lysate supernatant; Lanes 3-5: washes with 20 mM, 40 mM, and 60 mM imidazole, respectively; Lane 6: nickel-affinity chromatography filler; Lanes 7-8: eluates with 300 mM and 500 mM imidazole, respectively.

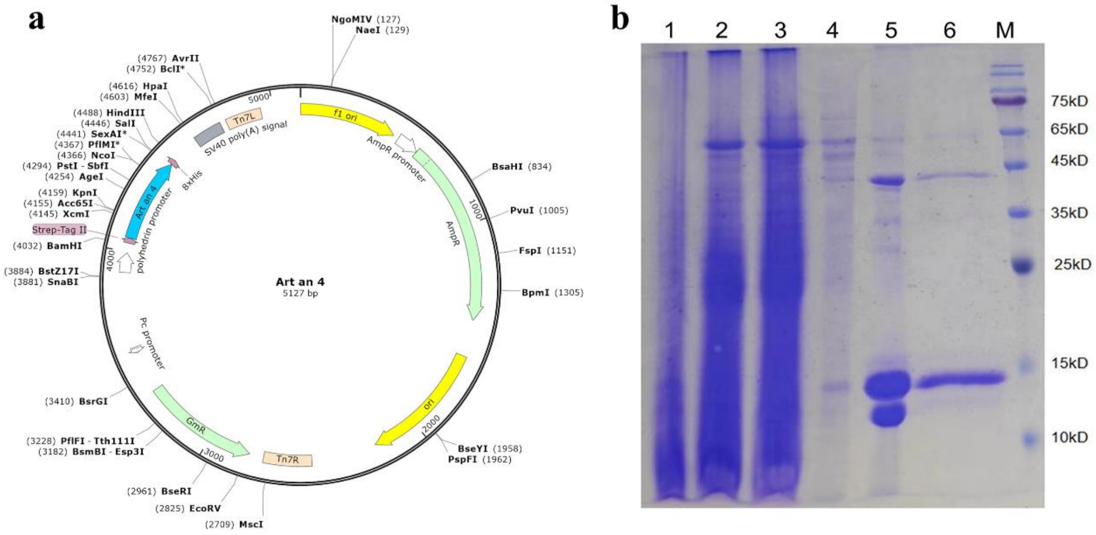

Fig. S3 Purification of Art an 4 from *Artemisia* pollen extract. (a) pFastBac-Art an 4 recombinant plasmid. (b) Affinity Chromatography analysis of recombinant protein Art an 4. Lane M, standard marker; Lane 1: cell lysate precipitate; Lane 2: cell lysate supernatant; Lane 3, the flow-through fraction; Line 4, wash fraction collected from cells using wash buffer; Lane 5, nickel-affinity chromatography filler; Lane 6, eluate obtained from cells with elution buffer.

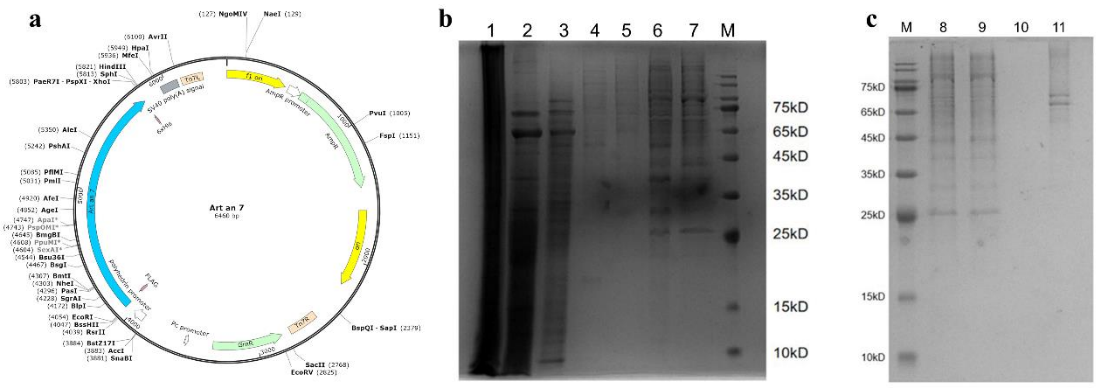

Fig. S4 Purification of Art an 7 from *Artemisia* pollen extract. (a) pFastBac-Art an 7 recombinant plasmid. (b) First Affinity Chromatography analysis of recombinant protein Art an 7. Lane M, standard marker; Lane 1: cell lysate precipitate; Lane 2: cell lysate supernatant; Lane 3-5, washes with 10 mM, 25 mM, and 50 mM imidazole, respectively; Line 6, nickel-affinity chromatography filler; Lane 7, eluates with 250 mM imidazole. (c) Second Affinity Chromatography analysis of recombinant protein Art an 7 with 250 mM imidazole in Elute buffer. Lane M, standard marker; Lane 8, flow-through without passage through the nickel column; Lane 9: flow-through after passage through the nickel column; Lane 10: wash fraction collected from cells using wash buffer; Lane 11: eluate obtained from cells with elution buffer.
