# Supplementary material for: Evolution of IgE sensitization profiles to Artemisia pollen allergens in the allergic pediatric population
Source: World Allergy Organ J. 2026 Apr 29;19(5):101391. doi: 10.1016/j.waojou.2026.101391 (PMC13141757; doi:10.1016/j.waojou.2026.101391)
Supplement: Multimedia component 2 [file mmc2.pdf]

Table S1 Clinical characteristics of enrolled patients in the study

| Patients, no. | Gender | Age | Diagnosis | Total IgE (IU/mL) | Specific IgE to Artemisia (IU/mL) |
|---|---|---|---|---|---|
| 1 | Male | 6 | AA | 84.93 | 63.39 |
| 2 | Male | 8 | AA | 305.33 | 52.35 |
| 3 | Male | 5 | AA | 107.21 | 85.67 |
| 4 | Male | 4 | AA | 110.92 | 36.33 |
| 5 | Female | 5 | AA | 500.00 | 64.02 |
| 6 | Female | 4 | AA | 440.79 | 200.00 |
| 7 | Female | 2 | AA | 109.93 | 95.89 |
| 8 | Male | 4 | AA | 89.23 | 88.88 |
| 9 | Female | 3 | AA | 500.00 | 31.70 |
| 10 | Male | 4 | AA | 300.70 | 95.54 |
| 11 | Male | 5 | AA | 426.04 | 200.00 |
| 12 | Male | 10 | AA | 500.00 | 200.00 |
| 13 | Male | 4 | AA | 252.07 | 200.00 |
| 14 | Male | 4 | AA | 114.11 | 200.00 |
| 15 | Female | 6 | AA | 204.29 | 200.00 |
| 16 | Male | 10 | AA | 503.55 | 200.00 |
| 17 | Male | 8 | AA | 189.09 | 56.34 |
| 18 | Female | 3 | AA | 415.52 | 40.99 |
| 19 | Male | 11 | AA | 126.77 | 69.80 |
| 20 | Male | 9 | AR | 193.37 | 200.00 |
| 21 | Female | 6 | AR | 221.68 | 0.42 |

| | | | | | |
|---|---|---|---|---|---|
| 22 | Male | 4 | AR | 85.63 | 88.45 |
| 23 | Male | 5 | AR | 500.00 | 200.00 |
| 24 | Female | 7 | AR | 111.46 | 2.26 |
| 25 | Male | 12 | AA; AC | 109.30 | 54.25 |
| 26 | Female | 5 | AR | 500.00 | 200.00 |
| 27 | Male | 2 | AR | 171.68 | 67.74 |
| 28 | Male | 4 | AR | 163.24 | 80.09 |
| 29 | Female | 5 | AR | 125.32 | 150.39 |
| 30 | Male | 5 | AA | 149.33 | 200.00 |
| 31 | Male | 13 | AA; Urticaria | 195.57 | 76.30 |
| 32 | Male | 3 | AR | 83.48 | 0.42 |
| 33 | Female | 2 | AR; AC | 500.00 | 67.06 |
| 34 | Male | 3 | AA | 112.63 | 0.41 |
| 35 | Male | 2 | AR; Eczema | 102.60 | 200.00 |
| 36 | Male | 3 | AR; Urticaria | 202.95 | 2.19 |
| 37 | Male | 10 | AA; Urticaria | 500.00 | 200.00 |
| 38 | Male | 3 | AR | 144.56 | 0.61 |
| 39 | Female | 8 | AR | 128.68 | 0.70 |
| 40 | Female | 10 | AR | 370.03 | 29.08 |
| 41 | Male | 4 | AR | 179.99 | 117.27 |
| 42 | Male | 5 | AA | 500.00 | 200.00 |
| 43 | Male | 3 | AR | 115.65 | 81.57 |
| 44 | Female | 5 | AA; Urticaria | 79.23 | 50.00 |
| 45 | Male | 4 | AR | 99.93 | 100.46 |
| 46 | Female | 6 | AA | 522.50 | 200.00 |

| | | | | | |
|---|---|---|---|---|---|
| 47 | Male | 8 | AA | 500.00 | 0.55 |
| 48 | Male | 8 | AR | 76.86 | 0.59 |
| 49 | Male | 9 | AA | 73.64 | 2.34 |
| 50 | Female | 5 | AR | 521.95 | 63.88 |
| 51 | Female | 6 | AR | 31.01 | 0.59 |
| 52 | Male | 3 | AR | 89.04 | 26.54 |
| 53 | Female | 4 | AR | 103.75 | 10.03 |
| 54 | Male | 3 | AR | 35.87 | 5.34 |
| 55 | Male | 3.5 | AR | 96.96 | 5.50 |
| 56 | Male | 3 | AA | 114.89 | 0.70 |
| 57 | Male | 6 | AR | 110.47 | 64.75 |
| 58 | Male | 5 | AR | 101.41 | 22.98 |
| 59 | Female | 6 | AR; AC; Eczema | 113.42 | 2.03 |
| 60 | Female | 2 | AR; Urticaria | 365.36 | 104.59 |
| 61 | Male | 8 | AR; AC | 239.15 | 80.95 |
| 62 | Male | 4 | AR; AC | 117.47 | 27.47 |
| 63 | Male | 2 | AA | 522.50 | 109.99 |
| 64 | Female | 3 | AR; AC | 204.79 | 127.01 |
| 65 | Male | 7 | AA | 491.70 | 58.75 |
| 66 | Female | 9 | AR | 475.32 | 71.46 |
| 67 | Female | 7 | AR | 257.50 | 200.00 |
| 68 | Female | 3 | AR | 191.15 | 97.79 |
| 69 | Female | 3 | AR | 96.03 | 55.58 |
| 70 | Male | 12 | AR | 228.51 | 10.92 |
| 71 | Male | 2 | AR | 94.41 | 41.71 |

| | | | | | |
|---|---|---|---|---|---|
| 72 | Male | 8 | AR | 500.00 | 22.23 |
| 73 | Female | 9 | AR | 500.00 | 200.00 |
| 74 | Male | 10 | AA | 500.00 | 200.00 |
| 75 | Male | 6 | AR; Eczema | 500.00 | 136.90 |
| 76 | Female | 2 | AR | 500.00 | 15.76 |
| 77 | Male | 4 | AR | 120.55 | 17.11 |
| 78 | Male | 2 | AR | 500.00 | 46.19 |
| 79 | Male | 3 | AR; AC | 101.72 | 43.54 |
| 80 | Male | 4 | AR | 115.30 | 44.51 |
| 81 | Male | 2 | AR | 500.00 | 200.00 |
| 82 | Male | 3 | AA | 207.87 | 71.57 |
| 83 | Male | 3 | AR | 246.08 | 30.09 |
| 84 | Male | 3 | AR; AC | 169.69 | 0.43 |
| 85 | Male | 6 | AR | 500.00 | 148.40 |
| 86 | Female | 6 | AR | 347.74 | 1.24 |
| 87 | Male | 2 | AR | 373.03 | 200.00 |
| 88 | Male | 5 | AR; AC | 135.60 | 1.94 |
| 89 | Female | 3 | AA | 102.00 | 0.48 |
| 90 | Male | 6 | AA | 500.00 | 200.00 |
| 91 | Male | 2 | AR | 83.14 | 81.62 |
| 92 | Female | 10 | AR; AC | 294.38 | 200.00 |
| 93 | Female | 11 | AR; AC | 203.49 | 200.00 |
| 94 | Female | 5 | AR; AC | 500.00 | 1.92 |
| 95 | Male | 3 | AR | 142.38 | 24.63 |
| 96 | Male | 4 | AR | 104.84 | 11.79 |

| | | | | | |
|---|---|---|---|---|---|
| 97 | Male | 4 | AR; AC | 41.30 | 56.02 |
| 98 | Male | 3 | AR | 500.00 | 18.17 |
| 99 | Male | 5 | AR | 60.26 | 0.77 |
| 100 | Male | 2 | AR | 102.36 | 2.07 |
| 101 | Male | 8 | AA; AC | 198.26 | 51.17 |
| 102 | Male | 10 | AR; AC; Eczema | 500.00 | 200.00 |
| 103 | Male | 7 | AR | 110.17 | 7.21 |
| 104 | Male | 7 | AR | 262.44 | 16.34 |
| 105 | Male | 4 | AR | 133.29 | 38.11 |
| 106 | Male | 4 | AR | 240.73 | 22.60 |
| 107 | Male | 4 | AR | 80.10 | 13.66 |
| 108 | Female | 8 | AR | 95.71 | 27.56 |
| 109 | Female | 3 | AR | 74.45 | 9.21 |
| 110 | Female | 4 | AR | 64.76 | 0.61 |
| 111 | Female | 6 | AR | 521.48 | 51.35 |
| 112 | Male | 3 | AR; AC | 112.68 | 116.23 |
| 113 | Male | 4 | AR | 243.21 | 12.88 |
| 114 | Female | 3 | AR | 500.00 | 11.76 |
| 115 | Male | 3 | AR | 197.25 | 0.67 |
| 116 | Female | 2 | AR; AC | 450.46 | 15.42 |
| 117 | Male | 5 | AR; AC | 84.59 | 7.08 |
| 118 | Female | 4 | AR | 193.19 | 34.06 |
| 119 | Male | 4 | AA; AC | 152.47 | 12.65 |
| 120 | Male | 3 | AR | 104.90 | 94.14 |
| 121 | Female | 4 | AR | 210.02 | 200.00 |

| | | | | | |
|---|---|---|---|---|---|
| 122 | Male | 9 | AR | 428.71 | 200.00 |
| 123 | Male | 3 | AR | 53.06 | 3.12 |
| 124 | Female | 5 | AR | 85.23 | 8.31 |
| 125 | Male | 13 | AR | 231.17 | 104.66 |
| 126 | Male | 3 | AR; Urticaria | 236.24 | 158.21 |
| 127 | Female | 2 | AR; AC | 182.53 | 60.01 |
| 128 | Male | 3 | AR | 218.47 | 39.67 |
| 129 | Female | 9 | AR | 103.37 | 200.00 |
| 130 | Female | 8 | AR; Urticaria | 339.54 | 20.60 |
| 131 | Male | 7 | AA | 75.14 | 0.35 |
| 132 | Male | 2 | AR | 85.74 | 17.16 |
| 133 | Female | 5 | AR; AC | 113.45 | 1.58 |
| 134 | Female | 3 | AR | 197.82 | 99.03 |
| 135 | Male | 3 | AR | 72.89 | 0.37 |
| 136 | Female | 4 | AR | 101.94 | 7.41 |
| 137 | Male | 3 | AR | 56.22 | 169.30 |
| 138 | Female | 4 | AR | 194.81 | 59.47 |
| 139 | Male | 4 | AR | 141.30 | 45.97 |
| 140 | Male | 6 | AR | 251.08 | 200.00 |
| 141 | Female | 8 | AR | 152.19 | 85.75 |
| 142 | Male | 4 | AR; AC | 522.50 | 200.00 |
| 143 | Male | 6 | AR; AC | 129.41 | 200.00 |
| 144 | Female | 6 | AR | 500.00 | 200.00 |
| 145 | Female | 8 | AR | 522.5 | 84.77 |
| 146 | Female | 11 | AA | 194.33 | 200.00 |

| | | | | | |
|---|---|---|---|---|---|
| 147 | Male | 2 | AR | 110.19 | 91.29 |
| 148 | Male | 2 | AR | 56.68 | 0.42 |
| 149 | Female | 4 | AR | 256.36 | 12.92 |
| 150 | Male | 3 | AR | 50.27 | 4.81 |
| 151 | Male | 3 | AR | 404.78 | 75.49 |
| 152 | Female | 5 | AR | 84.83 | 25.89 |
| 153 | Male | 2 | AR | 104.46 | 3.49 |
| 154 | Female | 4 | AR | 105.56 | 1.55 |
| 155 | Male | 9 | AR | 500.00 | 200.00 |
| 156 | Female | 5 | AR | 76.14 | 1.12 |
| 157 | Female | 10 | AR | 66.10 | 2.07 |
| 158 | Female | 4 | AR; Urticaria | 105.04 | 19.39 |
| 159 | Male | 6 | AR | 59.16 | 27.95 |
| 160 | Female | 7 | AR | 101.77 | 10.21 |
| 161 | Male | 3 | AR; AC | 74.04 | 75.93 |
| 162 | Female | 5 | AR | 115.83 | 3.23 |
| 163 | Male | 9 | AR | 108.51 | 27.01 |
| 164 | Male | 4 | AR; AC | 193.42 | 53.68 |
| 165 | Female | 9 | AR | 229.83 | 200.00 |
| 166 | Male | 4 | AR; AC | 273.72 | 200.00 |
| 167 | Female | 5 | AA | 178.53 | 105.56 |
| 168 | Male | 5 | AR; AC | 195.3 | 95.18 |
| 169 | Male | 5 | AR | 108.57 | 19.12 |
| 170 | Male | 4 | AR | 186.16 | 56.20 |
| 171 | Male | 6 | AR | 180.10 | 25.54 |

| | | | | | |
|---|---|---|---|---|---|
| 172 | Male | 3 | AR | 123.73 | 200.00 |
| 173 | Female | 4 | AR | 500.00 | 68.86 |
| 174 | Female | 8 | AR | 408.26 | 200.00 |
| 175 | Male | 5 | AR | 230.92 | 14.81 |
| 176 | Male | 4 | AR | 127.53 | 12.77 |
| 177 | Female | 3 | AR | 188.44 | 2.25 |
| 178 | Male | 3 | AR | 60.75 | 1.17 |
| 179 | Female | 11 | AR; Eczema | 451.32 | 200.00 |
| 180 | Male | 2 | AA; AC | 21.58 | 0.35 |
| 181 | Male | 11 | AR; AC | 114.74 | 28.36 |
| 182 | Female | 4 | AR | 23.23 | 0.46 |
| 183 | Female | 3 | AR; AC | 101.33 | 51.11 |
| 184 | Female | 4 | AR | 81.47 | 0.41 |
| 185 | Male | 2 | AR | 240.61 | 200.00 |
| 186 | Male | 3 | AR | 273.04 | 200.00 |
| 187 | Female | 5 | AR | 191.46 | 91.13 |
| 188 | Male | 2 | AR | 341.78 | 90.47 |
| 189 | Female | 4 | AR | 29.13 | 0.36 |
| 190 | Male | 8 | AR | 186.62 | 200.00 |
| 191 | Male | 3 | AR | 99.29 | 200.00 |
| 192 | Male | 8 | AR; Eczema | 78.56 | 49.78 |
| 193 | Male | 6 | AR | 111.72 | 65.43 |
| 194 | Male | 9 | AR | 103.26 | 36.71 |
| 195 | Female | 2 | AR; AC | 59.14 | 0.35 |
| 196 | Female | 5 | AA | 9.36 | 52.23 |

Abbreviations: AR, allergic rhinitis; AA, allergic asthma; AC, allergic conjunctivitis

Table S2 Prevalence of allergen positivity by gender and age group

| Allergen positive no. (%) | Gender | | | Age | | | |
|---|---|---|---|---|---|---|---|
| | Male (n=123) | Female (n=73) | *P*-value | ≤3 (n=63) | 4-6 (n=84) | 7-14 (n=49) | *P*-value |
| Tree pollen (Cypress, Elm, Sycamore, Willow, Poplar) | 66(53.66) | 36(49.32) | 0.556 | 32(50.79) | 43(51.19) | 27(55.10) | 0.884 |
| Mold (*Penicillium notatum, Aspergillus fumigatus, Cladosporium, Alternaria, Rhizopus, Mucor*) | 38(30.89) | 17(23.29) | 0.252 | 18(28.57) | 26(30.95) | 11(22.45) | 0.571 |
| Animal dander (Dog, Cat) | 30(24.39) | 17(23.29) | 0.861 | 18(28.57) | 16(19.05) | 13(26.53) | 0.363 |
| House dust mite (House dust mite, *Dermatophagoides pteronyssinus*) | 1(0.81) | 1(1.37) | 1.000 | 1(1.59) | 1(1.19) | 0(0.00) | 1.000 |
| House dust | 11(8.94) | 3(4.11) | 0.204 | 5(7.94) | 5(5.95) | 4(8.16) | 0.826 |
| Milk | 19(15.45) | 13(17.81) | 0.665 | 12(19.05) | 16(19.05) | 4(8.16) | 0.203 |
| Beef and Lamb | 4(3.25) | 3(4.11) | 0.713 | 2(3.17) | 3(3.57) | 2(4.08) | 1.000 |
| Egg fractions | 6(4.88) | 3(4.11) | 1.000 | 6(9.52) | 2(2.38) | 1(2.04) | 0.122 |
| Nut (Peanut, Pistachio, Cashew, Hazelnut) | 4(3.25) | 1(1.37) | 0.653 | 2(3.17) | 1(1.19) | 2(4.08) | 0.613 |

| | | | | | | | |
|---|---|---|---|---|---|---|---|
| Fruit (Mango, Pineapple, Apple, Peach, Strawberry) | 1(0.81) | 2(2.74) | 0.557 | 0(0.00) | 2(2.38) | 1(2.04) | 0.612 |
| Shellfish (Shrimp, Crab, Scallop) | 2(1.63) | 0(0.00) | 0.530 | 0(0.00) | 0(0.00) | 2(4.08) | 0.062 |
| Marine fish (Cod, Salmon, Bass) | 1(0.81) | 2(2.74) | 0.557 | 0(0.00) | 2(2.38) | 1(2.04) | 0.612 |
